# Treatment-Free Remission in Chronic Myeloid Leukemia Patients Treated With Low-Dose TKIs: A Feasible Option Also in the Real-Life. A Campus CML Study

**DOI:** 10.3389/fonc.2022.839915

**Published:** 2022-03-03

**Authors:** Alessandra Iurlo, Daniele Cattaneo, Silvia Artuso, Dario Consonni, Elisabetta Abruzzese, Gianni Binotto, Monica Bocchia, Massimiliano Bonifacio, Fausto Castagnetti, Sara Galimberti, Antonella Gozzini, Miriam Iezza, Roberto Latagliata, Luigiana Luciano, Alessandro Maggi, Maria Cristina Miggiano, Patrizia Pregno, Giovanna Rege-Cambrin, Sabina Russo, Anna Rita Scortechini, Agostino Tafuri, Mario Tiribelli, Carmen Fava, Gianantonio Rosti, Robin Foa, Massimo Breccia, Giuseppe Saglio

**Affiliations:** ^1^Hematology Division, Foundation IRCCS Ca’ Granda Ospedale Maggiore Policlinico, Milan, Italy; ^2^Department of Oncology and Hemato-Oncology, University of Milan, Milan, Italy; ^3^Epidemiology Unit, Foundation IRCCS Ca’ Granda Ospedale Maggiore Policlinico, Milan, Italy; ^4^Hematology Division, Sant’Eugenio Hospital, Rome, Italy; ^5^Department of Medicine, Hematology and Clinical Immunology, Padua School of Medicine, Padua, Italy; ^6^Hematology Unit, Azienda Ospedaliera Universitaria Senese, Siena, Italy; ^7^Department of Medicine, Section of Hematology, University of Verona, Verona, Italy; ^8^Institute of Hematology “L. and A. Seràgnoli”, Department of Experimental, Diagnostic and Specialty Medicine, University of Bologna, “S. Orsola-Malpighi” Hospital, Bologna, Italy; ^9^Department of Clinical and Experimental Medicine, University of Pisa, Pisa, Italy; ^10^Division of Hematology, AOU Careggi, Firenze, Italy; ^11^Division of Hematology, Belcolle Hospital, Viterbo, Italy; ^12^Division of Hematology, Department of Clinical Medicine and Surgery, Federico II University, Napoli, Italy; ^13^Division of Hematology, Hospital “S.G. Moscati”, Taranto, Italy; ^14^Division of Hematology, San Bortolo Hospital, Vicenza, Italy; ^15^Division of Hematology, AOU Città della Salute e della Scienza, Torino, Italy; ^16^Division of Internal Medicine and Hematology, San Luigi Gonzaga Hospital, Turin, Italy; ^17^Division of Hematology, Dipartimento di Patologia Umana dell’Adulto e dell’Età Evolutiva, Policlinico G. Martino, University of Messina, Messina, Italy; ^18^Division of Hematology, Department of Molecular and Clinical Sciences, Polytechnic University of Marche, Ancona, Italy; ^19^Division of Hematology, Azienda Ospedaliera Universitaria Sant’Andrea, Rome, Italy; ^20^Division of Hematology and BMT, Department of Medical and Morphological Research, University of Udine, Udine, Italy; ^21^Department of Clinical and Biological Sciences, University of Torino, Torino, Italy; ^22^Scientific Direction, IRCCS Istituto Romagnolo per lo Studio dei Tumori (IRST) “Dino Amadori”, Meldola, Italy; ^23^Division of Hematology, Department of Precision and Translational, Policlinico Umberto 1, Sapienza University, Rome, Italy

**Keywords:** chronic myeloid leukemia, tyrosine kinase inhibitors (TKI), treatment-free remission (TFR), low dose, adverse event (AE), real life

## Abstract

Treatment-free remission (TFR) has become a primary therapeutic goal in CML and is also considered feasible by international guidelines. TKIs dose reduction is often used in real-life practice to reduce adverse events, although its impact on TFR is still a matter of debate. This study aimed to explore the attitude of Italian hematologists towards prescribing TKIs at reduced doses and its impact on TFR. In September 2020, a questionnaire was sent to 54 hematology centers in Italy participating to the Campus CML network. For each patient, data on the main disease characteristics were collected. Most of the hematologists involved (64.4%) believed that low-dose TKIs should not influence TFR. Indeed, this approach was offered to 194 patients. At the time of TFR, all but 3 patients had already achieved a DMR, with a median duration of 61.0 months. After a median follow-up of 29.2 months, 138 (71.1%) patients were still in TFR. Interestingly, TFR outcome was not impaired by any of the variables examined, including sex, risk scores, BCR-ABL1 transcript types, previous interferon, type and number of TKIs used before treatment cessation, degree of DMR or median duration of TKIs therapy. On the contrary, TFR was significantly better after dose reduction due to AEs; furthermore, patients with a longer DMR duration showed a trend towards prolonged TFR. This survey indicates that low-dose TKI treatment is an important reality. While one third of Italian hematologists still had some uncertainties on TFR feasibility after using reduced doses of TKIs outside of clinical trials, TFR has often been considered a safe option even in patients treated with low-dose TKIs in the real-life setting. It should be noted that only 28.9% of our cases had a molecular recurrence, less than reported during standard dose treatment. Consequently, TFR is not impaired using low-dose TKIs.

## Introduction

Small molecule tyrosine kinase inhibitors (TKIs) targeting *BCR-ABL1* have dramatically improved the outcome of patients with chronic myeloid leukemia (CML). Indeed, when TKI therapy is addressed appropriately, it can lead to an optimal molecular response in most cases and a life expectancy approaching that of age-matched individuals in the general population (1). However, lifelong TKI therapy can have consequences, including chronic, mostly low-grade, adverse events (AEs) that can negatively impact patients’ quality of life (2, 3), adherence to therapy and, finally, on the success of treatment (4, 5). For these reasons, in real-life practice, TKI dose reductions are often required to reduce AEs (6, 7).

In recent years, treatment-free remission (TFR) has become a new therapeutic goal and is considered feasible even by international expert guidelines. According to European LeukemiaNet (ELN) recommendations, some aspects are mandatory (e.g. CML in chronic phase [CP], access to high-quality RT-qPCR, rapid response of PCR results), some are minimal (first-line therapy or second-line if intolerant to first-line; typical b2a2 or b3a2 *BCR-ABL1* transcript type; TKI treatment duration of at least 5 years for imatinib and 4 years for the second-generation TKIs) and some are optimal (deep molecular response [DMR] duration longer than 3 years if the patient is in sustained MR4.0 or 2 years if MR4.5) (8, 9). The Gruppo Italiano Malattie EMatologiche dell’Adulto (GIMEMA) CML Working Party has also recently developed a project aimed at selecting the treatment policies that can increase the probability of achieving TFR. In detail, a consensus was reached on the assessment of disease risk, first-line treatment choice, and the definition of responses requiring a TKI change, with the primary goal of optimizing the treatment strategy for TFR (10).

However, the likelihood of achieving and maintaining TFR depends on several factors that may be leukemia- or patient-related, including persistence of CD26+ leukemic stem cells (11) or patients’ immunological competence (12–15).

Most TFR-designed clinical trials and real-life experiences have shown this approach to be safe in CML patients who have gained a sustained DMR during treatment at a standard dose and most patients abruptly discontinue TKI treatment (16–25).

More recently, some authors have attempted a gradual treatment withdrawal prior to TKI discontinuation as part of clinical trials. Specifically, in the DESTINY study, the aim was to examine a TKI de-escalation treatment in patients with a stable major molecular response (MMR) or MR4, evaluated by at least three tests over 12 months, after a minimum of three years’ treatment with standard doses of imatinib, nilotinib, or dasatinib (26). In all 174 enrolled patients, no progression or cytogenetic relapse was reported, and the monthly monitoring quickly identified the 12 patients who lost MMR; they resumed full-dose therapy, and all regained an MMR within 4 months. None of these patients developed *BCR-ABL1* point mutations due to the reduced TKI doses. Furthermore, chronic AEs improved in most cases (27).

The ongoing DANTE trial is a prospective, single arm, phase II study aimed at assessing the effect of nilotinib reduced to half the standard dose during a 12-month consolidation period on TFR in CML-CP patients treated first-line with nilotinib who reached a sustained DMR prior to study entry. The primary endpoint is the percentage of patients in full TFR 96 weeks after the start of the consolidation phase. During the TFR phase, a loss of MMR requires resuming treatment with nilotinib at 300 mg BID (28).

In a more recent French real-life retrospective study that included 77 CML patients, 26 subjects were managed with low-dose TKIs before stopping treatment. Interestingly, a higher percentage of successful TFR was observed at 12 or 60 months in the cohort receiving lower dose TKIs (80.8% *vs.* 56.8% at 12 months and 58.8% *vs.* 47.5% at 60 months). Median time to MMR loss in the whole cohort was 6.2 months and all patients rapidly re-achieved an MMR after resuming TKI therapy (29).

In a subsequent review of real-life practice of patients receiving low-dose TKIs after the achievement of an MMR, due to intolerable AEs, 76 patients eventually discontinued low-dose TKIs and 2-year TFR rate in these patients was 74.1% (30).

The aim of the present study was to explore the attitude of Italian hematologists towards the use of reduced dose TKIs and its impact on TFR attempts in a large series of CML-CP patients.

## Materials and Methods

In September 2020, an *ad hoc* questionnaire was sent to the 54 hematology centers in Italy participating to the Campus CML network. This interactive network connects many Italian hematology specialists active in the field of CML with the aim of sharing experiences and updates for the diagnosis and treatment of the disease, the identification and prevention of specific toxicities of the drugs used and on possible future therapeutic approaches (31, 32).

As reported in the **Supplementary Material**, this online survey consisted of two parts: the first was questions regarding the use of reduced doses of TKIs in clinical practice and the propensity of physicians to offer TFR even to subjects treated with reduced doses of TKI for AEs or to patients with many comorbidities after achieving an optimal molecular response.

Subsequently, each participating center was asked to provide data on key disease characteristics for each patient who attempted TFR after low-dose TKI treatment. They included: socio-demographic variables, risk scores (i.e. Sokal and ELTS), all treatments (including prior interferon) before and after discontinuation, duration of each treatment, reasons for dose reduction, and best response to each treatment.

Molecular monitoring and ranking of responses were defined according to current ELN recommendations (8, 33–37): in particular, DMR as MR4 (*BCR-ABL1* ratio ≤0.01% with at least 10.000 ABL1 copies), MR4.5 (*BCR-ABL1* ratio ≤0.0032% with at least 32.000 ABL1 copies), or MR5 (*BCR-ABL1* ratio ≤0.001% with at least 100.000 ABL1 copies).

Follow-up information was updated in May 2021.

We analyzed the time to molecular relapse (truncated to four years) based on selected variables using the Kaplan-Meier estimator and the log-rank-test. Statistical analyses were performed with Stata 16 (StataCorp. 2019).

## Results

### Low-Dose TKIs in Real-Life

The survey was completed by 45 (83.3%) of centers involved. All reported having CML patients in treatment with low-dose TKIs [total number = 1.785 out of 5.637 (31.7%) regularly followed CML-CP patients]. More specifically, a reduction in TKI dose occurred in 24.4% of patients treated with imatinib, 27.8% with nilotinib, 35.3% with dasatinib, 73.7% with bosutinib and 75.4% with ponatinib.

### Low-Dose TKIs and TFR Feasibility

Most Italian hematologists (64.4%) believed that the use of reduced doses of TKIs should not preclude attempts at TFR; in fact, this approach was offered to 194/1.785 (10.9%) patients, whose demographics and clinical baseline data are shown in **Table 1**. The dose reduction was due to AEs in more than half of the cases (109 patients, 56.2%), with 42 (21.7%) patients (24 in first line, 13 in second line, and 5 in third line) who still obtained a DMR despite TKI de-escalation. In the remaining patients TKIs were de-escalated after achieving the optimal molecular milestone (**Table 2**).

**Table 1:** Baseline demographics and clinical characteristics of CML patients.

Characteristics	Patients (N = 194)
**M/F**	91/103
**Age at CML diagnosis** (years), median (range)	49.7 (19.1 – 79.8)
**Sokal score**	
Low, n (%)	100 (51.6)
Intermediate, n (%)	55 (28.3)
High, n (%)	28 (14.4)
NA, n (%)	11 (5.7)
**ELTS score**	
Low, n (%)	145 (74.7)
Intermediate, n (%)	25 (12.9)
High, n (%)	12 (6.2)
NA, n (%)	12 (6.2)
***BCR-ABL1* p210 transcript type**	
e14a2, n (%)	116 (59.8)
e13a2, n (%)	49 (25.3)
**Previous IFN**, n (%)	35 (18.0)
**Time from diagnosis to TKI discontinuation** (months), median (range)	120.4 (26.3 – 355.9)
**Duration of TKI therapy** (months), median (range)	118.1 (25.7 – 235.6)
**Duration of low-dose TKI** (months), median (range)	35.9 (0.6 – 194.9)
**Duration of DMR before TKI discontinuation** (months), median (range)	61.0 (4.0 – 180.0)
**Best response at time of dose reduction**, n (%)	
CHR	4 (2.1)
CCyR	9 (4.6)
MMR	32 (16.5)
DMR	149 (76.8)
**MR at TKI discontinuation**, n (%)	
MMR	3 (1.6)
DMR	191 (98.4)
MR4	44 (22.7)
MR4.5	79 (40.7)
MR5	68 (35.0)

CML, chronic myeloid leukemia; NA, not available; IFN, interferon; CHR, complete hematological response; CCyR, complete cytogenetic response; MMR, major molecular response; DMR, deep molecular response; MR, molecular response.

**Table 2:** Reasons for dose reduction according to each TKI.

	First-line(N=116)	Second-line(N=63)	Third-line or later(N=15)	Overall(N=194)
Imatinib, n (AEs/MR)	47/32	3/0	0/1	50/33
Nilotinib, n (AEs/MR)	12/15	16/19	5/2	33/36
Dasatinib, n (AEs/MR)	6/4	12/10	4/0	22/14
Bosutinib, n (AEs/MR)	–	–	2/0	2/0
Ponatinib, n (AEs/MR)	–	1/2	1/0	2/2

AEs, adverse events; MR,molecular response.

The TKI most frequently used at a reduced dose prior to the TFR attempt was imatinib (83 patients out of 717 – 11.6%), followed by nilotinib (69 patients out of 344 – 20.1%), dasatinib (36 patients out of 322 – 11.2%) and, to a lesser extent, ponatinib (four patients out 184 – 2.2%) and bosutinib (two patients out of 218 – 0.9%). Overall, 105 (54.1%) patients experienced a ≥50% reduction in TKI dose from baseline (**Table 3**). Prior to attempting TFR, 116 (59.8%) patients had received only one TKI, while 63 and 15 patients were treated in second or subsequent lines of therapy, respectively, with a median duration of TKI treatment of 118.1 months (range, 25.7-235.6). At the time of TFR, all but three patients (98.4%) had already achieved a DMR, with a median DMR duration of 61.0 months (range, 4.0-180.0).

**Table 3:** Treatment characteristics.

	Patients (N = 194)
Medication at onset of TKI discontinuation, n (%)
Imatinib	83 (42.8)
Nilotinib	69 (35.6)
Dasatinib	36 (18.6)
Bosutinib	2 (1.0)
Ponatinib	4 (2.0)
**Dosage of imatinib before treatment cessation**, n (%)	
300 mg/d	52 (62.6)
200 mg/d	31 (37.4)
**Dosage of nilotinib before treatment cessation**, n (%)	
200 mg/d	6 (8.7)
300 mg/d	21 (30.5)
400 mg/d	19 (27.5)
450 mg/d	12 (17.4)
600 mg/d	11 (15.9)
**Dosage of dasatinib before treatment cessation**, n (%)	
20 mg/d	4 (11.1)
50 mg/d	20 (55.6)
80 mg/d	12 (33.3)
**Dosage of bosutinib before treatment cessation**, n (%)	
400 mg/d	2 (100)
**Dosage of ponatinib before treatment cessation**, n (%)	
15 mg/d	3 (75.0)
15 mg every other day	1 (25.0)

### TFR Outcome

**Figure 1:**
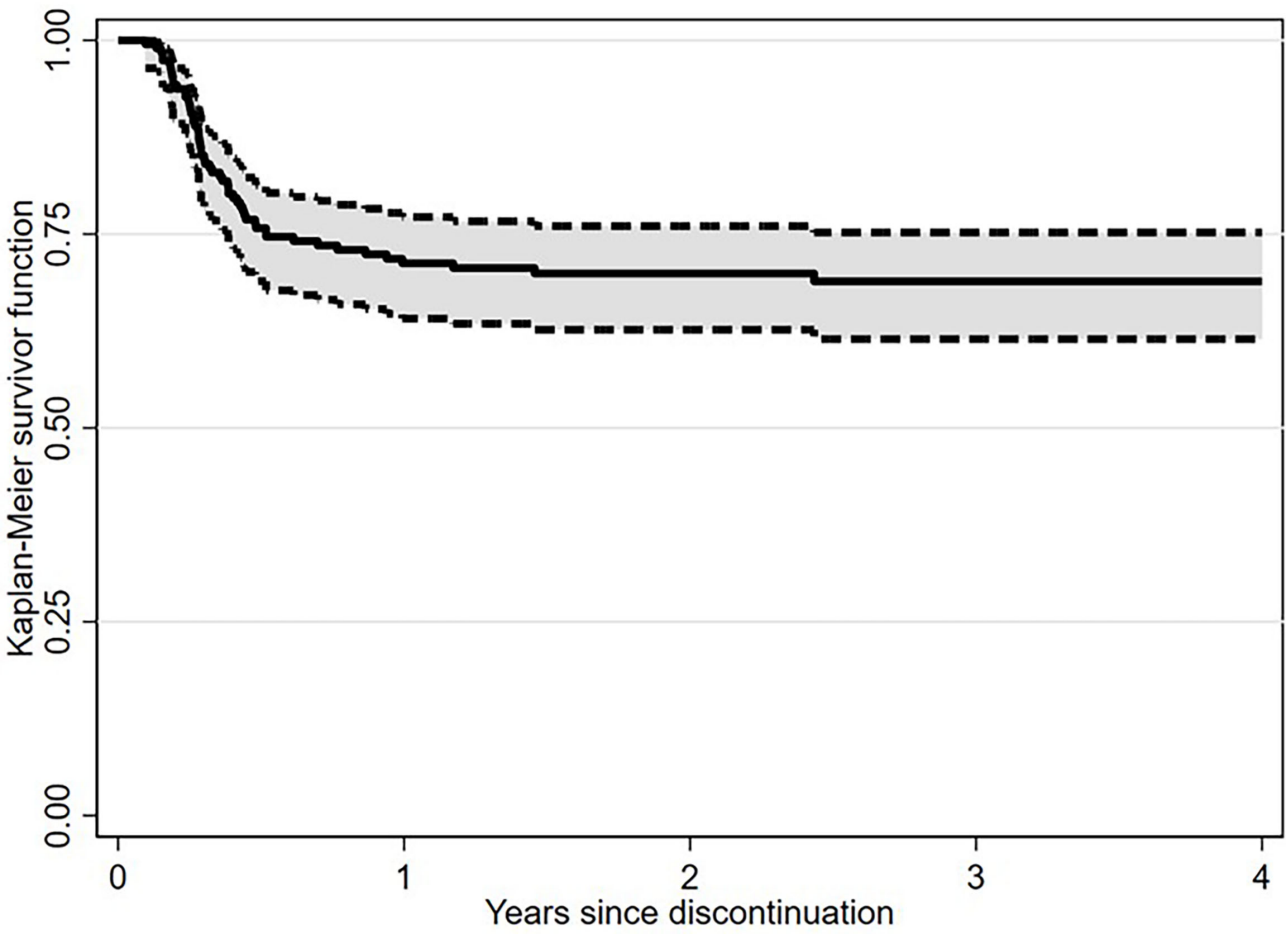
Treatment-free remission (TFR) after tyrosine kinase inhibitor (TKI) therapy (Kaplan-Meier estimate).

**Figure 2:**
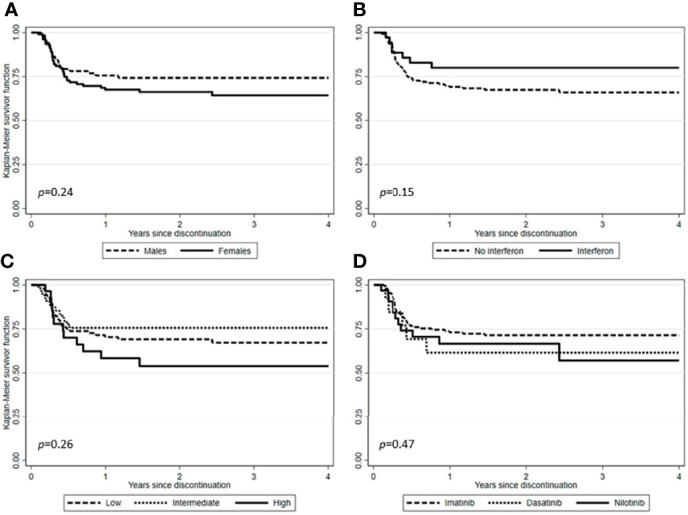
Treatment-free remission (TFR) after tyrosine kinase inhibitor (TKI) therapy (Kaplan-Meier estimate). **(A)** TFR according to sex. **(B)** TFR according to pretreatment with interferon. **(C)** TFR according to Sokal risk score. **(D)** TFR according to TKIs used before therapy cessation.

**Figure 3:**
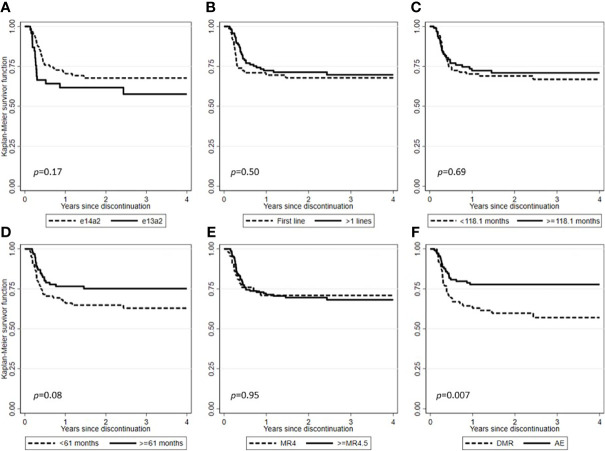
Treatment-free remission (TFR) after tyrosine kinase inhibitor (TKI) therapy (Kaplan-Meier estimate). **(A)** TFR according to *BCR-ABL1* transcript type. **(B)** TFR according to lines of therapy. **(C)** TFR according to median duration of TKIs therapy. **(D)** TFR according to median duration of DMR. **(E)** TFR according to the degree of DMR. **(F)** TFR according to the main reasons for TKIs dose reduction.

After a median follow-up from TKI discontinuation of 29.2 months (range, 1.7-128.7), 138 (71.1%) patients were still in TFR (**Figure 1**). Interestingly, in patients experiencing a TKI dose reduction, TFR outcome was not affected by any of the variables examined, including sex, Sokal or ELTS risk scores, *BCR-ABL1* transcript types, prior interferon therapy, type and number of TKIs used before treatment cessation, degree of DMR (i.e., MR4 *vs.* MR4.5 or better) or median duration of TKIs therapy (**Figures 2** and **3**). On the contrary, TFR was significantly better after dose reduction due to AEs (*vs.* cases with dose de-escalation after DMR achievement); furthermore, patients with a longer DMR duration (i.e., >61 months) showed a trend towards prolonged TFR (**Figure 3**).

### Molecular Recurrence

Median time to molecular recurrence (≥MMR) in the whole cohort was 3.6 months (range, 1.2-29.6), with 45/56 (80.4%) patients experiencing early relapse (within 6 months). In detail, one molecular recurrence (33.3%) was recorded in three patients who discontinued low-dose TKIs in MMR and 55 molecular recurrences (28.8%) in 191 patients who discontinued therapy in DMR. All the cases that experienced a molecular recurrence resumed the same dose of TKI treatments ongoing before the TFR attempt. After a median time from TKI resumption of 7.8 months (range, 1.0-37.3), MMR or DMR was re-achieved in all cases, with no difference between first- and second- or third-generation TKIs (7.0 *vs.* 7.9 months).

## Discussion

Evidence is accumulating that TKI dose modifications are feasible during treatment of CML and are an important consideration for the prevention and management of AEs, improving adherence and reducing treatment interruptions, and enabling high rates of DMR to be achieved (98.4% in our series) (38). Furthermore, dose optimization together with the prevention of AEs could also pursue the goal of achieving and maintaining an optimal molecular response, as trials of intermittent TKI treatment have shown that responding patients are often overtreated. Indeed, in the Italian INTERIM study, older CML patients (>65 years) treated with imatinib 400 mg/day 1-month on and 1-month off were able not only to maintain an MMR in most cases but sometimes also to improve the depth of their molecular response (39, 40).

It should also be stressed that a TKI dose optimization should be considered in advance. Indeed, once chronic toxicities develop, the beneficial effect of this approach is still a matter of debate, especially in some specific contexts (41).

Using a mathematical model, Fassoni et al. reported for the first time strong evidence suggesting that for most patients who have already achieved a sustained remission, a reduction in TKI dose of at least 50% does not lead to a reduction in long-term treatment efficacy and maintains a secondary decline in *BCR-ABL1* levels (42).

Similarly, second-generation TKIs have been shown to maintain their efficacy profile when used at a lower dose by reducing AEs: in particular, dasatinib 50 mg once daily proved to be effective and safe as initial therapy for CML-CP patients, showing 12-month MMR and DMR rates of 79% and 46%, respectively (43, 44). In the NILO-RED study, after achieving MMR on the standard nilotinib schedule, patients were switched to a low-dose regimen as maintenance therapy, providing preliminary evidence that switching to nilotinib once daily maintenance is feasible and safe, regardless of previous therapies (45).

Considering bosutinib, the original first-line study of this drug in newly diagnosed CML-CP patients failed to achieve its primary endpoint of demonstrating a superior cytogenetic response to imatinib, partly due to the AEs profile at the recommended dose of 500 mg once daily (46). However, the subsequent BFORE trial, using a lower bosutinib dose (400 mg once daily) in the same clinical setting, showed its improved efficacy compared to imatinib, with a better tolerability profile than the 500 mg daily dose (47).

The question of a direct relationship between the doses of TKIs and their efficacy/safety profile is of fundamental importance with regard to ponatinib: in fact, data from both clinical trials and real-life experiences led to the indication to reduce its daily dose in patients who have already achieved at least an MCyR (48, 49). Furthermore, considering the high anti-leukemic potency expressed by ponatinib, it is conceivable that lower-dose regimens or full-dose induction followed by dose reduction may also be useful for intolerant patients (50, 51).

Treatment discontinuation is now a primary therapeutic goal and appears to be a safe option for about half of patients who achieve an optimal response (52). However, most TFR clinical trials have demonstrated that this approach is safe in CML patients who have gained a sustained DMR during treatment at a standard dose and no systematic assessment of long-term TKI dose de-escalation has been performed, particularly in the real-life setting.

With this in mind, we then explored the impact of low-dose TKIs on TFR feasibility in a large series of CML-CP patients managed in Italy.

In this study, treatment with low-dose TKIs after achievement of molecular milestones represented a frequent clinical practice performed by Italian hematologists for CML patients, the main reason being the reduction in the risk of AEs. Bosutinib and ponatinib were in fact the most frequently used drugs at reduced dosages compared to those officially registered, both to prevent AEs and due to patients in more advanced therapeutic lines.

The dose reduction practice, even more so if due to AEs (**Figure 3**), did not prejudice the possibility of obtaining a DMR and therefore attempting a treatment discontinuation. However, this chance is feasible only in a selected subset of patients, probably related to a less aggressive disease. Indeed, not only most patients did not lose the molecular response achieved after the TKI dose reduction, but 21.7% of those not in DMR at the time of dose reduction were able to achieve it later.

While being aware of the limitations of this study, mainly represented by its retrospective nature, we would like to emphasize that only 28.9% of the patients in our series experienced a molecular recurrence, less than reported in patients treated at a standard dose. Notably, in a recent Italian collection of 293 CML-CP patients who discontinued TKI while in DMR, overall estimated TFR was 62% after a median follow-up of 34 months, which is well below of 71.1% reported in this study, with a comparable median time to restart treatment of six months (21).

The most likely explanation for this is that patients who have been able to maintain DMR after dose reduction are a select population more likely to undergo long term and successful TFR, as patients who have not been able to maintain DMR are excluded as they had to return to a full dosage treatment. In this sense the practice of dose reduction before total TKI discontinuation will probably not increase the absolute number of CML patients who can reach a successful TFR, but it can at least decrease the percentage of those who, after a complete stop of TKI therapy, can undergo the stressful need to restart therapy.

## Data Availability Statement

The original contributions presented in the study are included in the article/**Supplementary Material**. Further inquiries can be directed to the corresponding author.

## Ethics Statement

Ethical review and approval was not required for the study on human participants in accordance with the local legislation and institutional requirements. Written informed consent for participation was not required for this study in accordance with the national legislation and the institutional requirements.

## Author Contributions

AI conceptualized and designed the study. AI, DCa, SA, EA, GB, MoB, MaB, FC, SG, AG, MI, RL, LL, AM, MM, PP, GR-C, SR, AS, AT, MT, CF, GR, RF, MBr, and GS collected and assembled the data. DCo performed the statistical analysis. AI and DCa wrote the manuscript. RF, MBr, and GS critically reviewed the manuscript. AI, DCa, SA, DCo, EA, GB, MoB, MaB, FC, SG, AG, MI, RL, LL, AM, MM, PP, GR-C, SR, AS, AT, MT, CF, GR, RF, MBr, and GS approved the final version of the manuscript.

## Funding

The only funds used were provided by the authors’ institutions.

## Conflict of Interest

The authors declare that the research was conducted in the absence of any commercial or financial relationships that could be construed as a potential conflict of interest.

## Publisher’s Note

All claims expressed in this article are solely those of the authors and do not necessarily represent those of their affiliated organizations, or those of the publisher, the editors and the reviewers. Any product that may be evaluated in this article, or claim that may be made by its manufacturer, is not guaranteed or endorsed by the publisher.
