# Supplementary material for: Treatment-Free Remission in Chronic Myeloid Leukemia Patients Treated With Low-Dose TKIs: A Feasible Option Also in the Real-Life. A Campus CML Study
Source: Front Oncol. 2022 Mar 3;12:839915. doi: 10.3389/fonc.2022.839915 (PMC8927081; doi:10.3389/fonc.2022.839915)
Supplement: Supplementary file 1 [file DataSheet_1.doc]

**CAMPUS CML SURVEY: LOW-DOSE TKIs AND TFR**

1) Do you follow CML patients treated with low-dose TKIs? **❑** YES **❑** NO

2) If YES, indicate the absolute number and percentage of patients on reduced dose therapy compared to the total number of patients for each TKI.

**❑** IMATINIB Number:___________ Percentage:_________%

**❑** NILOTINIB Number:___________ Percentage:_________%

**❑** DASATINIB Number:___________ Percentage:_________%

**❑** BOSUTINIB Number:___________ Percentage:_________%

**❑** PONATINIB Number:___________ Percentage:_________%

3) If YES, please provide the reason for reduced dosage:

**❑** IMATINIB

**❑ **Intolerance/Toxicity Number:___________ Percentage:_________%

**❑ **Comorbidities Number:___________ Percentage:_________%

**❑** MR achievement Number:___________ Percentage:_________%

**❑ **Other:_____________ Number:___________ Percentage:_________%

**❑** NILOTINIB

**❑ **Intolerance/Toxicity Number:___________ Percentage:_________%

**❑ **Comorbidities Number:___________ Percentage:_________%

**❑** MR achievement Number:___________ Percentage:_________%

**❑ **Other:_____________ Number:___________ Percentage:_________%

**❑** DASATINIB

**❑ **Intolerance/Toxicity Number:___________ Percentage:_________%

**❑ **Comorbidities Number:___________ Percentage:_________%

**❑** MR achievement Number:___________ Percentage:_________%

**❑ **Other:_____________ Number:___________ Percentage:_________%

**❑** BOSUTINIB

**❑ **Intolerance/Toxicity Number:___________ Percentage:_________%

**❑ **Comorbidities Number:___________ Percentage:_________%

**❑** MR achievement Number:___________ Percentage:_________%

**❑ **Other:_____________ Number:___________ Percentage:_________%

**❑** PONATINIB

**❑ **Intolerance/Toxicity Number:___________ Percentage:_________%

**❑ **Comorbidities Number:___________ Percentage:_________%

**❑** MR achievement Number:___________ Percentage:_________%

**❑ **Other:_____________ Number:___________ Percentage:_________%

4) Was the reduced dosage adopted from the start of treatment?

**❑ **YES, for the following reason(s):**____________________________**

**❑ **NO, the drug was started at the standard dosage and reduced only subsequently for the reasons indicated in point 3

5) In which line(s) of treatment did you usually prescribe low-dose TKIs?

**❑ **first line

**❑ **second line

**❑ **third or subsequent line

6) Do you think that TKIs dose reduction could jeopardize a possible subsequent attempt at TFR?

**❑ **YES

**❑ **NO

7) Did you refer patients treated with low-dose TKIs to TFR outside of clinical trials?

**❑ **YES

**❑ **NO

8) If YES, which was the low-dose TKI used prior to TFR?

**❑** IMATINIB Number:___________ Percentage:_________%

**❑** NILOTINIB Number:___________ Percentage:_________%

**❑** DASATINIB Number:___________ Percentage:_________%

**❑** BOSUTINIB Number:___________ Percentage:_________%

**❑** PONATINIB Number:___________ Percentage:_________%

9) If YES, how many TKIs had the patients taken before attempting at TFR?

**❑** 1 Number:___________ Percentage:_________%

**❑** 2 Number:___________ Percentage:_________%

**❑** 3 or more Number:___________ Percentage:_________%
